# The Anticonvulsant Ethosuximide Disrupts Sensory Function to Extend *C. elegans* Lifespan

**DOI:** 10.1371/journal.pgen.1000230

**Published:** 2008-10-24

**Authors:** James J. Collins, Kimberley Evason, Christopher L. Pickett, Daniel L. Schneider, Kerry Kornfeld

**Affiliations:** Department of Developmental Biology, Washington University School of Medicine, St. Louis, Missouri, United States of America; The Jackson Laboratory, United States of America

## Abstract

Ethosuximide is a medication used to treat seizure disorders in humans, and we previously demonstrated that ethosuximide can delay age-related changes and extend the lifespan of the nematode *Caenorhabditis elegans*. The mechanism of action of ethosuximide in lifespan extension is unknown, and elucidating how ethosuximide functions is important for defining endogenous processes that influence lifespan and for exploring the potential of ethosuximide as a therapeutic for age-related diseases. To identify genes that mediate the activity of ethosuximide, we conducted a genetic screen and identified mutations in two genes, *che-3* and *osm-3*, that cause resistance to ethosuximide-mediated toxicity. Mutations in *che-3* and *osm-3* cause defects in overlapping sets of chemosensory neurons, resulting in defective chemosensation and an extended lifespan. These findings suggest that ethosuximide extends lifespan by inhibiting the function of specific chemosensory neurons. This model is supported by the observation that ethosuximide-treated animals displayed numerous phenotypic similarities with mutants that have chemosensory defects, indicating that ethosuximide inhibits chemosensory function. Furthermore, ethosuximide extends lifespan by inhibiting chemosensation, since the long-lived *osm-3* mutants were resistant to the lifespan extension caused by ethosuximide. These studies demonstrate a novel mechanism of action for a lifespan-extending drug and indicate that sensory perception has a critical role in controlling lifespan. Sensory perception also influences the lifespan of *Drosophila*, suggesting that sensory perception has an evolutionarily conserved role in lifespan control. These studies highlight the potential of ethosuximide and related drugs that modulate sensory perception to extend lifespan in diverse animals.

## Author Summary

Aging is a major factor that contributes to disease and disability in humans, but no medicines have been demonstrated to delay human aging. We previously conducted a screen for FDA-approved drugs that can extend the lifespan of the nematode worm *C. elegans*, resulting in the identification of ethosuximide, a medicine used to treat epilepsy. To elucidate the mechanism of action of ethosuximide in lifespan extension, we conducted a genetic screen for *C. elegans* mutations that cause resistance to ethosuximide. Here, we describe the identification of genes that are critical for ethosuximide sensitivity. These genes are necessary for the function of neurons that mediate sensory perception. Furthermore, ethosuximide treatment caused defects in sensory perception. These results indicate that ethosuximide affects lifespan by inhibiting neurons that function in the perception of sensory cues. These studies highlight the importance of sensory neurons in lifespan determination and demonstrate that a drug can act on specific cells within the nervous system to extend lifespan. Sensory perception also modulates *Drosophila* lifespan, suggesting this is an evolutionarily conserved relationship. Our results indicate that sensory perception may be a promising target for pharmacological extension of lifespan in a variety of animals.

## Introduction

Pharmacological compounds that extend lifespan could delay the progression of age-related degenerative changes and age-related illnesses such as Alzheimer's disease and cardiovascular disease. In addition, the characterization of drugs that extend lifespan can elucidate endogenous mechanisms involved in lifespan determination, since the targets of these drugs are likely to influence normal aging. The short lifespan and rapid aging of invertebrates makes them powerful models for the identification of drugs that extend lifespan and for the characterization of the mechanism of action of these drugs [1]–[3]. The free-living soil nematode *Caenorhabditis elegans* has been a leading system for studying genetic and pharmacologic influences on lifespan. Four categories of compounds have been reported to extend *C. elegans* lifespan: a variety of antioxidant compounds [4]–[9]; complex mixtures derived from plants [10],[11]; resveratrol, a potential modulator of Sir2 activity [12],[13]; and medications such as heterocyclic anticonvulsant medications that may act by affecting neural activity [14]–[17]. Compounds that extend the lifespan of vertebrates have not been well characterized. However, a recent report showing that resveratrol can extend the lifespan of a short-lived fish suggests that compounds that extend invertebrate lifespan may be relevant to vertebrate biology [18].

By screening 19 drugs from different structural and functional classes that are FDA-approved for human use, Evason et al. (2005) discovered that ethosuximide can extend the lifespan of *C. elegans* [14]. Ethosuximide is a small, heterocyclic ring compound of the succinimde class that is approved for human use as an anticonvulsant [19]. Trimethadione is a structurally related anticonvulsant that is a member of the oxazolidinedione class, and trimethadione also extends *C. elegans* lifespan [14]. Ethosuximide is commonly used in clinical practice whereas trimethadione is rarely used due to the potential for adverse side effects. Ethosuximide extended the mean adult lifespan of wild-type animals grown on agar dishes by 17% [14]. The effect is dose dependent, and at high doses ethosuximide causes toxicity. In addition, ethosuximide extends the span of time that animals display fast body movement and pharyngeal pumping, demonstrating that ethosuximide delays age-related functional declines in addition to extending lifespan. Ethosuximide has been shown to affect the activity of multiple ion channels in vertebrate cultured cells, including T-type calcium channels [20]–[22]. The relationship between these activities in cultured cells and the anticonvulsant activity in whole animals has yet to be defined fully. Furthermore, the mechanism of action for lifespan extension in worms is not well characterized.

To elucidate the mechanism of action of ethosuximide, we conducted a genetic screen for mutations that cause resistance to the drug. Screening for drug resistance is a well-established approach in *C. elegans* [23]. A mutation can cause resistance to a drug for several different reasons such as altering the molecular target of the drug, the cellular target of the drug, or the metabolism of the drug. An example of resistant mutants that identified the molecular target include the ivermectin resistant locus *avr-15*, which encodes a glutamate gated chloride channel that binds ivermectin [24] and the **α**-amanitin resistant locus *ama-1*, which encodes a RNA polymerase that binds **α**-amanatin [25],[26]. An example of a gene that affects drug metabolism and was identified in a screen for drug resistant mutants is *nrf-6*, which functions in intestinal cells to promote fluoxetine sensitivity [27].

Here we analyze the mechanism of action of ethosuximide in lifespan extension and show that it is related to the activity of chemosensory neurons, indicating that these neurons are the cellular target of ethosuximide in lifespan extension. *C. elegans* and other animals live in complex environments that can change rapidly, and therefore these animals have evolved the ability to respond quickly to changing conditions. The ability to perceive chemosensory cues and mount behavioral responses enables an animal to adjust to environmental changes. *C. elegans* uses ciliated chemosensory neurons located in the anterior and posterior of the animal to respond to numerous soluble and volatile cues [28]–[30]. Mutations that cause defects in cilia structure or sensory signaling within chemosensory neurons disrupt chemotaxis towards soluble and volatile chemicals [29]–[31]. These chemosensory neurons also influence adult lifespan, since mutations that cause defects in the structure of cilia or mutations that cause defects in sensory signaling can extend lifespan [32],[33]. Furthermore, laser ablation of the chemosensory neurons ASI, AWA, and AWC, separately or together, can increase adult lifespan [34]. These results indicate that the activity of certain chemosensory neurons promotes a short lifespan.

To characterize the mechanism of action of ethosuximide, we conducted a genetic screen and identified mutations in two genes, *che-3* and *osm-3*, that cause resistance to ethosuximide-mediated toxicity. Mutations in *che-3* and *osm-3* cause defects in overlapping sets of chemosensory neurons and can extend lifespan [32]. These findings indicate that ethosuximide extends lifespan by inhibiting a subset of chemosensory neurons. Here we present results that strongly support this model. Ethosuximide treated wild-type animals displayed numerous phenotypic similarities with mutants that have chemosensory defects, indicating that ethosuximide inhibits chemosensory function. Importantly, the long-lived *osm-3* mutants did not respond to lifespan extending doses of ethosuximide. These studies demonstrate a novel mechanism of action for a lifespan extending drug and demonstrate the potential of pharmacologically targeting the sensory system as a means to extend animal lifespan.

## Results

### The *C. elegans* T-type Calcium Channel, CCA-1, Is Not Required for Ethosuximide to Extend Lifespan or Stimulate Egg-Laying

**Figure 1:**
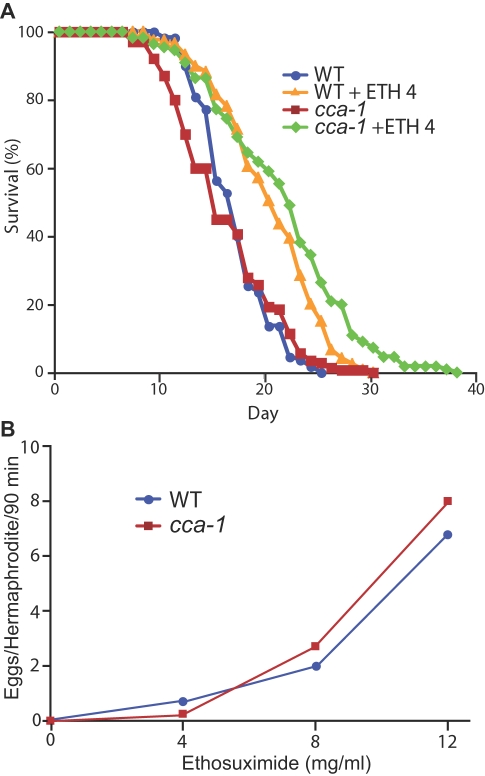
Ethosuximide treatment extended lifespan and stimulated egg laying in *cca-1* null-mutants. (A) Ethosuximide extended the lifespan of *cca-1(gk30)* mutants. Wild-type or *cca-1(gk30)* hermaphrodites were cultured with 0 mg/ml or 4 mg/ml ethosuximide (+ETH 4) from conception until death. The mean lifespans in days±SD for WT, WT+ETH 4, *cca-1*, and *cca-1*+ETH 4 were 16.1±3.3 (n = 110), 19.5±4.8 (n = 120), 15.2±4.9 (n = 140), and 20.8±6.6 (n = 110), respectively. The differences in mean lifespan of WT compared to WT+ETH 4 and *cca-1* compared to *cca-1*+ETH 4 were statistically significant, p<0.0001 (Student T-test). (B) *cca-1* mutants responded to ethosuximide stimulated egg-laying. The mean number of eggs laid±SD by wild-type animals (blue circles) was 0.0±0.0, 0.7±2.2, 2.0±2.5, and 6.8±5.2 for ethosuximide concentrations of 0, 4, 8, and 12 mg/ml, respectively. The mean number of eggs laid±SD by *cca-1(ad1650)* mutant animals (red squares) was 0.0±0.0, 0.25±1.0, 2.7±3.1 and 8.0±5.6 for ethosuximide concentrations of 0, 4, 8, and 12 mg/ml, respectively. n>23 for all trials. Differences in egg-laying between WT and *cca-1* at ethosuximide concentrations of 0, 4, 8 and 12 mg/ml were not statistically significant, p>0.05 (Student T-test).

Studies using vertebrate cultured cells have led to the proposal that T-type calcium channels may be the molecular target of ethosuximide in controlling seizures [20],[22],[35]. To determine if ethosuximide inhibits T-type calcium channels to extend lifespan, we analyzed a null mutation in the gene encoding the *C. elegans* orthologue of the mammalian T-type calcium channel, *cca-1(gk30)* [36],[37]. If ethosuximide inhibits T-type calcium channels to extend lifespan, then we predict that (1) a *cca-1* loss-of-function mutant will be long-lived and (2) a *cca-1* loss-of-function mutant will not respond to the lifespan extension caused by ethosuximide. *cca-1(gk30)* mutants displayed a mean adult lifespan of 15.2 days, which was not significantly different from the 16.1 day mean adult lifespan of wild-type animals (Figure 1A). *cca-1(gk30)* hermaphrodites treated with ethosuximide displayed a robust lifespan extension, indicating that ethosuximide does not require T-type calcium channels to extend *C. elegans* lifespan (Figure 1A).

In addition to extending lifespan, ethosuximide also stimulates the rate of egg-laying [14]. To determine if ethosuximide requires T-type calcium channels to stimulate egg-laying, we utilized a well-described assay that involves counting the eggs laid by a young adult hermaphrodite that is transferred from a Petri dish with abundant food to M9 liquid culture with no food [38]. We analyzed a second putative *cca-1* null allele, *cca-1(ad1650)* [36],[37]. Wild-type and *cca-1(ad1650)* hermaphrodites transferred to M9 buffer laid zero eggs in 90 minutes (Figure 1B), demonstrating that egg-laying is strongly inhibited by these conditions. Wild-type and *cca-1(ad1650)* hermaphrodites displayed a similar, dose-dependent increase in egg-laying in response to ethosuximide (Figure 1B), indicating that T-type calcium channels are not required for ethosuximide stimulated egg-laying. Collectively, these results suggest that ethosuximide modulates lifespan and egg-laying in *C. elegans* by acting on molecular targets distinct from the T-type calcium channel CCA-1.

### A Genetic Screen for Ethosuximide Resistant Mutants Implicates Ciliated Neuron Function in Ethosuximide Sensitivity

The identification and characterization of mutants that are resistant to the activity of a drug has been a useful approach for elucidating the mechanism of drugs in *C. elegans* [24], [39]–[41]. The analysis of candidate genes, including *cca-1* and genes that influence longevity [14], did not clearly identify the target of ethosuximide. Therefore, we conducted a genetic screen for mutations that cause resistance to ethosuximide. To identify a phenotype for conducting a genetic screen that is more suitable than lifespan, we performed a dose response analysis. The optimal dosage for lifespan extension by ethosuximide was 2–4 mg/ml in the culture media [14]. Concentrations of ethosuximide greater than 4 mg/ml caused dose dependent larval lethality (data not shown). The minimum dosage that caused fully penetrant lethality was 12 mg/ml ethosuximide. The larval lethal phenotype is ideal for the isolation of resistant mutants, because it is possible to screen large numbers of mutant animals for survival in 12 mg/ml ethosuximide. We mutagenized wild-type animals with EMS or ENU, screened approximately 200,000 haploid genomes for mutants that survived when cultured on 12 mg/ml ethosuximide, and isolated 48 mutants. Forty-two mutant strains were successfully backcrossed to wild-type animals, indicating that ethosuximide resistance in these strains is caused by a mutation in one gene. In all cases the mutant strains displayed partially penetrant survival in 12 mg/ml ethosuximide (Table 1 and data not shown), indicating that these mutations cause a shift in the dose response to ethosuximide and do not abrogate sensitivity to this drug. Here we describe six of these mutations that represent two complementation groups.

**Table 1:** Mutants with defects in cilia structure were resistant to ethosuximide toxicity.

Genotype	Amphid Dye-Filling∞	Percent Ethosuximide Resistant# (n)
WT	+	0 (783)
*che-3(am178)*	+	30.1 (199)
*che-3(am165)*	Dyf	26.1 (413)
*che-3(am162)*	Dyf	10.2 (525)
*che-3(e1124)*	Dyf†	30.8 (668)*
*osm-3(am161)*	Dyf	43.4 (426)
*osm-3(am177)*	Dyf	41.1 (451)
*osm-3(am172)*	Dyf	ND
*osm-3(p802)*	Dyf†	79.6 (621)
*che-13(p1805)*	Dyf†	62.0 (756)
*daf-10(e1387)*	Dyf†	22.6 (729)
*osm-5(p813)*	Dyf†	36.6 (209)*

**∞:** Animals cultured at 20°C were classified as normal (+) or defective (Dyf) for amphid neuron dye filling (see Materials and Methods).

# Experiments were done with 12 mg/ml ethosuximide. The differences in percent resistance between *che-3* alleles were not statistically significant except that *che-3(am162)* was significantly different from *che-3(am178)*, *che-3(am165)* and *che-3(e1124)* (p<0.0001). The differences in percent resistance between *osm-3* alleles were not statistically significant except that *osm-3(p802)* was significantly different from *osm-3(am161)* and *osm-3(am177) (p<0.0001)*.

**†:** The dye-filling phenotype of these mutants was described previously [28].

*: These values may underestimate the fraction of animals that were resistant to ethosuximide, since we observed multiple animals that were mature, indicating they were ethosuximide resistant, but they were desiccated on the side of the dish and not included in the data. We have observed that mutants with severe chemotaxis defects have a propensity to leave the agar surface.

**Figure 2:**
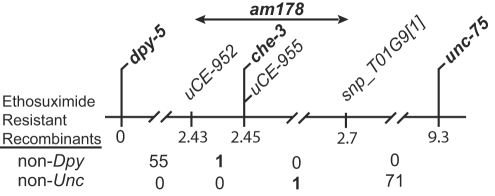
Positioning the *am178* mutation. The horizontal line represents a portion of Chromosome I. Genes that can be mutated to cause visible phenotypes and SNP markers are shown above; map units are shown below. From *dpy-5 am178*/CB4856 hermaphrodites, we selected 56 ethosuximide resistant, non-Dpy self-progeny. From *am178 unc-75*/CB4856 hermaphrodites, we selected 72 ethosuximide resistant, non-Unc self-progeny. An analysis of SNP markers in these strains positioned the recombination events in the intervals shown below. The double-headed arrow indicates a 250 kBp interval between the SNP markers *uCE-952* and *snp_T01G9* [*1*] that contains *am178*.

To characterize the mutations that cause ethosuximide resistance, we positioned these mutations on the *C. elegans* genetic and physical maps. The mutations *am178*, *am165*, and *am162* exhibited tight linkage to a single nucleotide polymorphism (SNP) marker positioned at the center of Chromosome I (see *Materials and Methods*). Three-factor mapping positioned *am178* between *dpy-5* and *unc-75* on Chromosome I (Figure 2). High resolution mapping positioned *am178* between SNP markers *uCE1-952* and *snp_T01G9* [*1*], a ∼250 kB interval on Chromosome I (Figure 2). To identify the gene affected by *am178*, we analyzed candidate genes in the interval by measuring the ability of existing mutations to cause resistance to ethosuximide lethality. To quantitatively assess the drug resistance of a mutant, we exposed embryos to 12 mg/ml ethosuximide for five to six days and measured the number of surviving animals that developed past the L1 larval stage. *che-3(e1124)* mutants displayed resistance to 12 mg/ml ethosuximide that was 31 percent penetrant (Table 1). Furthermore, *che-3(e1124)* failed to complement *am178* for resistance to ethosuximide, indicating that *am178* is a mutation in *che-3* (see *Materials and Methods*). To test the prediction that the *am178* mutation reduces the activity of the *che-3* gene, we generated transgenic *am178* mutants containing 32 kB of genomic DNA in a fosmid clone that included the *che-3* gene. This wild-type copy of the *che-3* gene rescued the *am178* mutant phenotype (see *Materials and Methods*). To identify the molecular lesion, we determined the DNA sequence of the predicted *che-3* exons and splice junctions using DNA from *am178* mutants. We identified a single base change that affects the splice junction following exon 9 (the 5′ splice site was changed from GTAT**G** in wild-type to GTAT**A** in *am178* mutants). Analysis of transcripts demonstrated that *am178* mutants produce an aberrantly spliced *che-3* mRNA that is predicted to encode a truncated CHE-3 protein, suggesting that *am178* is a loss-of-function mutation of the *che-3* gene.

**Figure 3:**
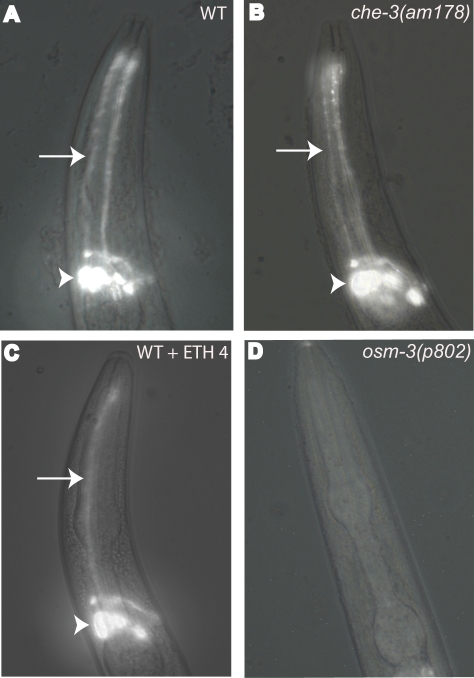
Ethosuximide treated animals and *che-3(am178)* mutants cultured at 20°C did not display dye-filling defects. Animals were incubated in the lipophilic dye DiO and observed using fluorescence microscopy at 200× magnification. Each panel illustrates the anterior tip of the animal (top) to the base of the bi-lobed pharynx (bottom). DiO stains amphid neuron processes (arrows) and cell bodies (arrowheads). Wild-type (WT) animals displayed robust staining that was not affected by treatment with 4 mg/ml ethosuximide (WT+ETH4). *che-3(am178)* mutants cultured at 20°C displayed robust staining, whereas *osm-3(p802)* mutants displayed no detectable staining (the Dyf phenotype).

*che-3* encodes a cytoplasmic dynein heavy-chain [42]. Dynein is a component of the intraflagellar transport (IFT) machinery, and this machinery builds and maintains the structure of ciliated neurons in *C. elegans*. Mutations that disrupt IFT components, such as CHE-3 dynein, result in defects in the structure of ciliated neurons [43]. *C. elegans* contains 60 ciliated neurons, and these neurons function in the perception of chemical and mechanical cues [28]–[31],[44]. The most extensively characterized ciliated neurons are present in the amphid organ, a bilaterally symmetrical neural structure located in the anterior of the animal. Some amphid neurons have ciliated dendrites that are exposed to the external environment, since they extend through a channel in the cuticle [28],[45]. Since these neurons are exposed to the environment, treating animals with lipophilic dyes such as DiO specifically stains these neurons (Figure 3A)[28],[46]. Mutants with severe defects in the structure of these neurons display no staining, a phenotype referred to as dye-filling defective (Dyf) (Figure 3D) [28],[31]. Many *che-3* mutations cause a robust Dyf phenotype [42].

To characterize *che-3(am178)* and two candidate *che-3* alleles that were positioned near the center of Chromosome I, *am162* and *am165*, we performed the dye-filling assay. *am162* and *am165* both caused a Dyf phenotype (Table 1), and both mutations failed to complement *che-3(e1124)* for dye-filling defects (*Materials and Methods*), indicating that *am162* and *am165* are alleles of *che-3*. *che-3(am178)* mutants cultured at 20°C displayed dye-filling that was not significantly different from wild-type (Table 1, Table 2 and Figure 3B). However, *che-3(am178)* mutants cultured at 27°C displayed a highly penetrant dye-filling defective phenotype (Table 2), indicating that the *am178* mutation partially reduces the activity of the *che-3* gene at the permissive temperature of 20°C and strongly reduces the activity of the *che-3* gene at the restrictive temperature of 27°C. To determine the temporal requirement for *che-3* gene activity, we cultured hermaphrodites at 20°C until the L4 stage of development and then shifted the animals to 27°C for 24 hours before scoring DiO staining. Using this regimen *che-3(am178)* mutants displayed a significant defect in DiO staining, indicating that *che-3* function is necessary after the L4 stage to maintain cilia structure (Table 2). The findings that *che-3(am178)* mutants cultured at 20°C displayed robust resistance to ethosuximide toxicity but did not display dye-filling defects indicate that gross morphological defects in amphid neuron structure are not required for *che-3* mutants to be resistant to ethosuximide.

**Table 2:** The *che-3(am178)* mutation caused temperature-sensitive defects in DiO staining of amphid neurons.

Genotype	Temperature (°C)∞	% Dyf	N
WT	20	0	22
*che-3(am178)*	20	1.3	76
WT	27	0	47
*che-3(am178)*	27	93	121
WT	20→20	0	64
*che-3(am178)*	20→20	0	83
WT	20→27	0	51
*che-3(am178)*	20→27	44	72

**∞:** For lines 1–4, hermaphrodites were cultured from conception to L4 at the indicated temperature. For lines 5–8, hermaphrodites were cultured from conception to L4 at 20°C and then shifted to the indicated temperature for approximately 24 hours.

### Three Mutations that Cause Ethosuximide Resistance Affect the *osm-3* Gene that is Necessary for Ciliated Neuron Structure

Since *che-3* mutations cause both ethosuximide resistance and DiO staining defects, we characterized dye-staining of the remaining ethosuximide resistant mutants. Three mutations that exhibited tightest linkage to a marker positioned at −3.7 on Chromosome IV, *am161*, *am172* and *am177*, caused defects in DiO staining (Table 1). We investigated the possibility that these are *osm-3* alleles because *osm-3* is located at −2.2 on Chromosome IV and can be mutated to produce defective DiO staining (Figure 3D). *am161*, *am172*, and *am177* each failed to complement the *osm-3* reference allele *p802* for DiO staining defects, suggesting that each mutation affects the *osm-3* gene (see *Materials and Methods*). Furthermore, the *osm-3(p802)* mutation caused 80 percent resistance to ethosuximide (Table 1), consistent with the model that *am161*, *am172*, and *am177* are *osm-3* alleles.

**Figure 4:**
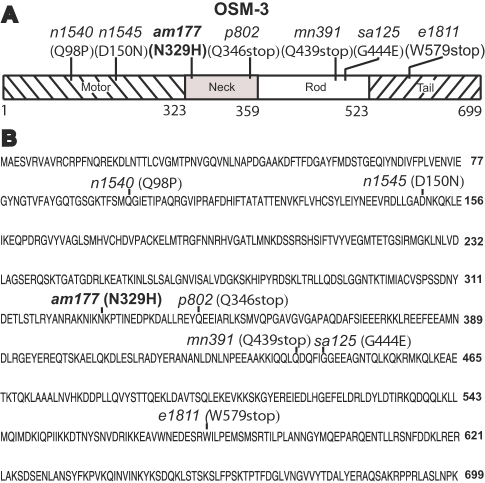
*am177* is a missense mutation of *osm-3*. (A) A schematic of the OSM-3 protein showing amino acid numbers (below) and four functional regions: the motor (left diagonal lines), neck (shaded), rod (open) and tail (right diagonal lines) [47]. The position of *am177* and six previously characterized mutations are shown above with the mutation name and molecular change [47]. (B) The predicted amino acid sequence of OSM-3 (M02B7.1B). The *am177* mutation changes codon 329 from AAC (N) to CAC (H), affecting the neck region of the protein.

Like the CHE-3 dynein, the OSM-3 kinesin is a component of the IFT machinery that is essential for the formation of ciliated nerve endings. The OSM-3 kinesin has been divided into four domains: motor, neck, rod, and tail (Figure 4) [47]. Previous molecular genetic studies identified two missense mutations that affect the motor region and one missense mutation that affects the rod region. To identify molecular lesions in the new *osm-3* alleles, we determined the DNA sequence of *osm-3* exons using DNA from *am177* mutants. We identified a single base change in the coding region of the *am177* allele; this missense mutation changes codon 329 from asparagine to histidine (Figure 4). The *am177* mutation in the *osm-3* gene is distinct from previously characterized missense mutations and affects the neck region, thus identifying a residue in the neck region that is critical for protein function (Figure 4).

### Chemosensory Neurons Are Necessary to Mediate Sensitivity to Ethosuximide

The demonstration that *che-3* and *osm-3* are necessary for sensitivity to ethosuximide-induced lethality suggests that the function of ciliated neurons is necessary to mediate the toxic effects of ethosuximide. To investigate this model, we exploited the well-characterized collection of mutations that affect ciliated neurons [28],[31]. *che-13*, *daf-10* and *osm-5* encode IFT components, and loss-of-function mutations of these genes cause robust defects in DiO staining. Loss-of-function mutations of *che-13*, *daf-10* and *osm-5* caused significant ethosuximide resistance of 62 percent, 23 percent, and 37 percent, respectively (Table 1). These results support the hypothesis that the function of ciliated neurons is necessary to mediate the effects of ethosuximide.

We identified five different genes (*che-3*, *osm-3*, *daf-10*, *osm-5*, *and che-13*) that influence ethosuximide toxicity. Because these genes encode diverse protein products, it is unlikely that these proteins are the direct molecular target of ethosuximide. However, these genes are all required for the structure or function of ciliated neurons, indicating that ciliated neurons may be an important cellular focus of ethosuximide function. The *che-3*, *che-13*, *daf-10*, and *osm-5* genes are necessary for the structure or activity of all 60 ciliated neurons [28]. By contrast, the *osm-3* gene is necessary for only a subset of ciliated neurons. *osm-3* expression is restricted to amphid, phasmid and inter labial neurons that function in chemosensation, and *osm-3* loss-of-function mutants are specifically defective in behaviors mediated by these neurons [28],[48],[49]. These observations define a subset of chemosensory neurons that are defective in *osm-3* mutants as critical for the response to ethosuximide toxicity.

### *osm-3* Mutants Responded to the Egg-Laying Stimulation Caused by Serotonin and Ethosuximide, Indicating that These Mutants Absorbed These Drugs

**Figure 5:**
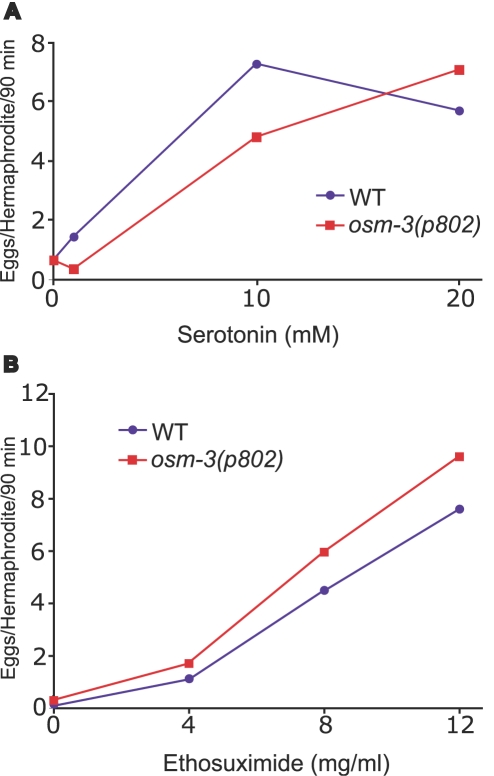
*osm-3(p802)* animals are sensitive to egg-laying stimulated by serotonin and ethosuximide. (A) Gravid hermaphrodites were placed individually in M9 buffer containing the indicated serotonin concentration, and the number of eggs laid in 90 minutes was scored. The mean number of eggs laid±SD by wild-type animals (blue circles) was 0.6±1.2, 1.4±1.8, 7.3±4.9, and 5.7±4.3 for serotonin concentrations of 0, 1, 10, and 20 mM, respectively. The mean number of eggs laid±SD by *osm-3(p802)* animals (red squares) was 0.6±1.7, 0.3±0.9, 4.8±3.5, and 7.1±3.4 for serotonin concentrations of 0, 1, 10, and 20 mM, respectively. N>11 for all trials. At all serotonin concentrations, differences in egg-laying between WT and *osm-3* were not statistically significant, *p>0.05* (Student t-test). (B) Experiments with ethosuximide were carried out as above with the indicated concentration of ethosuximide. The mean number of eggs laid±SD by wild-type animals (blue circles) was 0.2±0.5, 1.1±1.4, 4.5±4.2, and 7.6±3.6 for ethosuximide concentrations of 0, 4, 8, and 12 mg/ml, respectively. The mean number of eggs laid±SD by *osm-3* mutant animals (red squares) was 0.3±0.8, 1.7±2.6, 6.0±4.3, and 9.6±2.8 for ethosuximide concentrations of 0, 4, 8, and 12 mg/ml, respectively. n = 24 for all trials. Differences in egg-laying between WT and *osm-3* at ethosuximide concentrations of 0, 4, and 8 were not statistically significant, p>0.05 (Student T-test). At 12 mg/ml ethosuximide, *osm-3* animals laid significantly more eggs than wild-type animals, p = 0.03 (Student T-test).

In principle, mutations that disrupt cilium structure could cause resistance to ethosuximide toxicity because they affect the molecular target, the cellular target or the metabolism of the drug. Mutations that disrupt cilium structure prevent the lipophilic dye DiO from staining the membranes of ciliated neurons. This raises the possibility that these mutations also disrupt absorption of water soluble drugs, such as ethosuximide [24]. If this were the case then these mutants are predicted to be resistant to a wide variety of drugs. To investigate this possibility, we analyzed resistance to serotonin, a well-characterized compound that stimulates egg-laying [38]. We compared the resistance of wild-type hermaphrodites and *osm-3(p802)* mutants. *osm-3(p802)* has been extensively characterized using behavioral assays and electron microscopic analyses [28]. Genetic studies indicate that *osm-3(p802)* is a strong loss-of-function mutation, and molecular studies demonstrate that the allele contains a nonsense change at codon 346 [47] (Figure 4). Our analysis also supports the conclusion that *osm-3(p802)* is a strong loss-of-function mutation, since the *osm-3(p802)* mutation caused a significantly greater resistance to ethosuximide toxicity than the newly isolated *osm-3(am177)* and *osm-3(am161)* mutations. Wild-type animals and *osm-3(p802)* mutants displayed similar sensitivity to the stimulation of egg-laying caused by serotonin (Figure 5A). These results indicate that entry of serotonin into the animal is normal in *osm-3* mutants despite the defects in cilium structure.

To directly investigate the absorption of ethosuximide by cilium structure mutants, we analyzed a separate phenotype caused by ethosuximide. If drug absorption is mediated by ciliated neurons, then mutants with defective cilium structure are predicted to be resistant to all the effects of ethosuximide. By contrast, if drug absorption is not mediated by ciliated neurons, then mutants with defective cilium structure are predicted to be sensitive to the effects of ethosuximide mediated by non-ciliated cells. The ethosuximide effect we chose to analyze is the stimulation of egg-laying, since we previously showed this effect required the activity of non-ciliated HSN neurons that innervate the egg laying muscles [14]. We chose the *osm-3(p802)* mutant for these analyses because it has severe defects in cilium structure and is highly resistant to ethosuximide toxicity (Table 1). Wild-type and *osm-3* hermaphrodites transferred to M9 buffer laid an average of 0.17 and 0.29 eggs in 90 minutes, respectively (Figure 5B). Wild-type and *osm-3* hermaphrodites displayed a similar, dose-dependent increase in the rate of egg-laying in the presence of ethosuximide (Figure 5B), indicating that ethosuximide stimulates egg-laying in these conditions. These results demonstrate that *osm-3* mutants have a normal response to ethosuximide-stimulated egg-laying, indicating that the entry of ethosuximide into the animal is normal in these mutants.

### Mutations that Impair Ciliated Neurons and Treatment with Ethosuximide Cause Similar Defects in Chemotaxis

**Figure 6:**
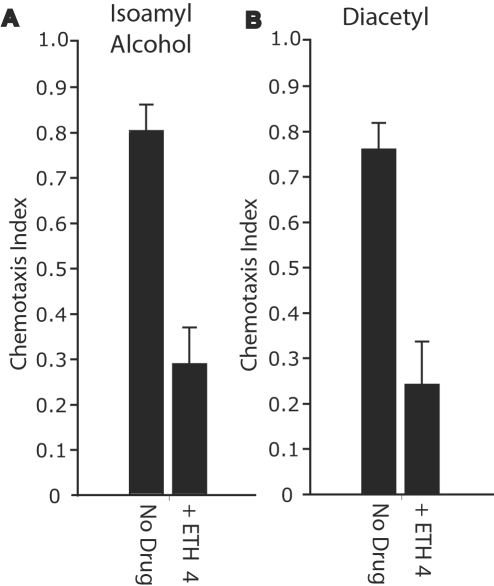
Ethosuximide disrupted chemotaxis toward the volatile attractants isoamyl alcohol and diacetyl. Wild-type animals grown from conception to adulthood on NGM plates containing 2 mg/ml ethosuximide or no drug were transferred to the center of a 10 cM chemotaxis plate containing 4 mg/ml of ethosuximide or no drug, respectively. A chemotaxis index (CI) to isoamyl alcohol or diacetyl was determined. (A) Chemotaxis to isoamyl alcohol. The mean CI±SD for untreated and ethosuximide treated animals was 0.8±0.2 and 0.3±0.2, respectively (p<0.0001, n = 8) (Student T-test). (B) Chemotaxis to diacetyl. The mean CI±SD for untreated and ethosuximide treated animals was 0.8±0.14 and 0.2±0.2, respectively (p = 0.0009, n = 6) (Student T-test). Error bars represent standard error of mean.

Our findings suggest that ethosuximide affects the activity of chemosensory neurons. To directly investigate this model, we compared the effects of ethosuximide treatment and mutations that cause defects in chemosensory neurons. Animals with defects in amphid neurons resulting from mutations or physical ablation do not respond properly to chemical cues from the environment and display defective chemotaxis [28]–[31]. To analyze how ethosuximide treatment affects chemotaxis, we measured chemotaxis towards the volatile odorant isoamyl alcohol which is mediated by AWC amphid neurons [29]. Untreated wild-type animals displayed a chemotaxis index towards isoamyl alcohol of about 0.8, and ethosuximide treatment significantly reduced this to about 0.3 (Figure 6A). This result indicates that ethosuximide disrupts the activity of AWC amphid neurons, resulting in defective chemotaxis.

To determine if ethosuximide treatment affects additional amphid neurons, we analyzed chemotaxis of ethosuximide treated animals to the volatile attractant diacetyl, an odorant detected by AWA amphid neurons [29]. Ethosuximide treatment significantly diminished chemotaxis toward diacetyl (Figure 6B), indicating that ethosuximide disrupts the activity of AWA neurons. These results suggest that treatment with ethosuximide disrupts multiple chemosensory amphid neurons that mediate chemotaxis, including AWC and AWA.

### Ethosuximide Treatment Caused Daf-c and Daf-d Phenotypes, Similar to Mutations that Disrupt Chemosensory Function

Amphid neurons play important roles in the developmental decision between forming an L3 larva that matures to a reproductive adult or a dauer larva that persists until environmental conditions improve. Laser ablation studies indicate that specific chemosensory amphid neurons are necessary to inhibit dauer formation (ASI, ADF, and ASG), and to promote dauer formation (ASJ) [50],[51]. Consistent with these observations, mutations that cause defects in multiple chemosensory neurons have complex effects on dauer formation. These mutations can cause dauer defective (Daf-d) phenotypes or dauer constitutive (Daf-c) phenotypes depending on the environmental conditions [32], [52]–[54]. To investigate the effects of ethosuximide on chemosensory neuron activity, we analyzed the effects of ethosuximide treatment on dauer formation in environmental conditions previously described to cause Daf-d and Daf-c phenotypes in animals with chemosensory neuron defects.

The combination of high temperature, high population density and low food availability promotes the formation of dauer larvae in wild-type animals [23]. Mutants with defects in chemosensory neurons display a Daf-d phenotype in this combination of culture conditions, indicating that these sensory neurons are necessary to respond to these environmental cues and promote dauer larvae formation [54]. To determine if ethosuximide treatment causes a similar Daf-d phenotype, we analyzed animals cultured at the high temperature of 25°C, at a high population density, and with low food availability. The *osm-3(p802)* mutation significantly reduced the average number of dauer larvae by 10-fold, from 250 to 26 (p<0.0001) (Table 3), consistent with previous observations [54]. Ethosuximide treatment of wild-type animals significantly reduced the average number of dauer larvae by 16-fold, from 250 to 16 (p<0.0001) (Table 3). These results indicate that ethosuximide inhibits chemosensory neurons that are necessary to promote dauer larvae formation.

**Table 3:** Ethosuximide inhibited dauer formation in a high-density, starved population at 25°C.

Genotype	Ethosuximide (mg/ml)	Dauer±SD (Number/Dish)	N (Dishes)
WT	0	250±110	13
WT	4	16±15***	11
*osm-3(p802)*	0	26±27***	10

***: p<0.0001, comparisons are to WT with no drug treatment. p values determined by Student T-test.

By contrast to the culture conditions described above, culturing animals in low density populations with abundant food at the extremely high temperature of 27°C reveals that chemosensory neuron function is necessary to inhibit dauer formation [32],[52]. To determine if ethosuximide treatment and mutations that disrupt chemosensory neurons cause similar defects, we analyzed dauer larvae formation at 27°C. The *osm-3(p802)* mutation significantly increased the fraction of dauer larvae from 0 to 7 percent (Table 4). Ethosuximide treatment of wild-type animals significantly increased the fraction of dauer larvae from 0 to 10 percent (p<0.0001) (Table 4), indicating that ethosuximide treatment and an *osm-3* mutation caused similar defects in the ability to inhibit dauer formation. To investigate the relationship between the dauer promoting effects of ethosuximide and the *osm-3(p802)* mutation, we treated *osm-3(p802)* mutants with ethosuximide. Ethosuximide treatment did not enhance the dauer arrest caused by *osm-3(p802)* (Table 4), suggesting that ethosuximide and *osm-3* mutations promote dauer arrest by a similar mechanism. Collectively, these results indicate that ethosuximide treatment can cause Daf-c and Daf-d phenotypes in specific environmental conditions, and this result supports the model that ethosuximide disrupts the activity of multiple chemosensory neurons.

**Table 4:** Ethosuximide stimulated dauer formation in a low-density, well-fed population at 27°C.

Genotype	Ethosuximide (mg/ml)	Dauer (%)	N (animals)
WT	0	0	1310
WT	4	9.9***	1322
*osm-3(p802)*	0	6.6	637
*osm-3(p802)*	4	5.0	697

***: p<0.0001, comparisons are to the same genotype with no drug treatment. p values determined by Student T-test.

### Mutations that Impair Chemosensory Neurons and Treatment with Ethosuximide Caused Similar Defects in L1 Arrest

Insulin/IGF-1 signaling is a critical regulator of dauer formation and an important modulator of adult lifespan [55]–[57]. *daf-2* encodes a receptor tyrosine kinase similar to the insulin receptor [56], and *age-1* encodes a phosphatidylinositol-3-kinase that functions downstream in the signaling pathway [58]. Loss-of-function mutations in *daf-2* and *age-1* promote dauer formation. In standard culture conditions that do not promote dauer formation, 2 mg/ml ethosuximide treatment enhanced the dauer arrest phenotype of *daf-2(m41)* mutants from 3.4 percent (N = 263) to 91.3 percent (N = 304), suggesting an interaction between ethosuximide and insulin/IGF-1 signaling.

In addition, the insulin/IGF-1 signaling pathway regulates the ability of animals that hatch in the absence of food to arrest at the L1 stage of development [59],[60]. Loss-of-function mutations in *daf-2* and *age-1* cause animals to arrest at the L1 stage inappropriately when food is present [55],[61]. The activity of chemosensory neurons modulates this L1 arrest, since mutations in genes such as *osm-3* and *che-3* enhance the L1 arrest phenotype of *daf-2* and *age-1* mutants [54],[61]. To test the effects of ethosuximide treatment on L1 arrest, we treated *daf-2* mutants with the lifespan-extending dose of 4 mg/ml ethosuximide. Treatment of *daf-2(e1370)* animals with ethosuximide significantly increased L1 arrest from 19.2 percent in untreated animals to 47.5 percent in drug treated animals (Table 5). The ability of mutations that affect chemosensory neurons to enhance the *daf-2* L1 arrest phenotype requires the activity of *daf-16*, since the addition of a *daf-16* mutation abrogates this effect [62]. Similarly, the *daf-16(mu86)* mutation significantly reduced the effect of ethosuximide treatment from 47.5 percent in *daf-2(e1370)* mutants to 2.3 percent in *daf-2(e1370)*; *daf-16(mu86)* mutants (Table 5). These results indicate that ethosuximide treatment and mutations that disrupt chemosensory neurons have a similar effect on the L1 arrest phenotype of an insulin-signaling mutant, consistent with the model that ethosuximide inhibits the activity of chemosensory neurons. One interpretation of these findings is that *daf-2(e1370)* mutants are hypersensitive to ethosuximide toxicity, since wild-type animals treated with 12 mg/ml ethosuximide also arrest development at the L1 stage.

**Table 5:** Ethosuximide enhanced *daf-2(e1370)* L1 arrest.

Genotype	Ethosuximide (mg/ml)	L1 arrest∞ (%)	N (animals)
WT	0	0	471
WT	4	0.6	449
*daf-2(e1370)*	0	19.2	332
*daf-2(e1370)*	4	47.5***	284
*daf-16(mu86)*	0	0	497
*daf-16(mu86)*	4	2.7***	558
*daf-2(e1370)daf-16(mu86)*	0	0.3	603
*daf-2(e1370)daf-16(mu86)*	4	2.3*	469

**∞:** This value includes arrested L1 larvae and eggs that failed to hatch, as described by Gems et al. (1998) [55]. Unhatched eggs were only a small proportion of this total. Statistical comparisons are to the same genotype with no drug treatment. Numbers with no asterisks are not significant (P>0.05); *, P<0.05; **, P<0.005; ***, P<0.0001. P values determined by Student T-test. The 47.5 value in line four is significantly larger than the 2.3 value in line eight (P<0.0001).

### *osm-3* Mutants Display an Extended Lifespan and Are Resistant to the Lifespan Extension Caused by Ethosuximide

**Figure 7:**
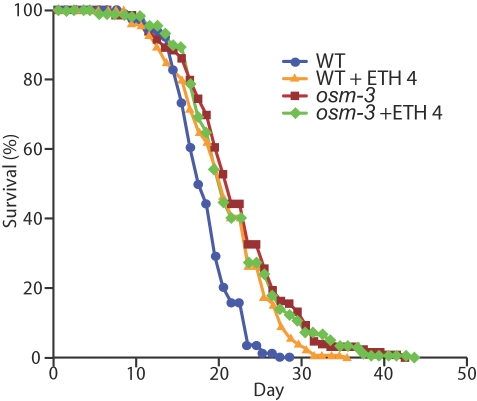
*osm-3* mutants were long-lived and failed to respond to lifespan extension by ethosuximide. Wild-type (WT) or *osm-3(p802)* hermaphrodites were cultured with 0 mg/ml or with 4 mg/ml ethosuximide (+ETH 4) from conception until death. Day 0 represents the L4 stage.

In addition to functioning in chemotaxis, dauer formation, and L1 larval arrest, chemosensory neurons play a role in adult lifespan determination [32]–[34]. The finding that ethosuximide treatment extends the adult lifespan and affects the activity of chemosensory neurons, suggests that ethosuximide extends adult lifespan by affecting the activity of chemosensory neurons. To investigate this model, we monitored the lifespan of *osm-3(p802)* hermaphrodites. *osm-3* mutants display a significantly extended lifespan [32] (Figure 7 and Table 6). If ethosuximide extends the adult lifespan by modulating the activity of chemosensory neurons, then *osm-3(lf)* animals are predicted to be resistant to the lifespan extension caused by ethosuximide. Treatment of wild-type hermaphrodites with 2 mg/ml or 4 mg/ml ethosuximide significantly extended the mean lifespan by 16 percent or 13 percent, respectively, and the maximum lifespan by 29 percent or 21 percent, respectively (Figure 7 and Table 6). By contrast, treatment of *osm-3(p802)* hermaphrodites with 2 mg/ml or 4 mg/ml ethosuximide did not cause a statistically significant extension of mean or maximum lifespan (Figure 7 and Table 6). These results support the model that ethosuximide extends lifespan by inhibiting chemosensory neurons.

**Table 6:** *osm-3* mutants displayed an extended lifespan and were resistant to the lifespan extension caused by ethosuximide.

Genotype	Ethosuximide (mg/ml)	Mean Lifespan±SD (days)	Percent Change∞	p∞	Maximum Lifespan±SD (days)#	Percent Change∞	p∞	N*
WT	0	18.7±3.7	-	-	24.9±1.3	-	-	164
	2	21.7±6.7	+16	<0.0001	32.2±2.4	+29.3	<0.0001	146
	4	21.2±5.6	+13.4	<0.0001	30.2±2.0	+21.3	<0.0001	158
*osm-3(p802)*	0	22.5±6.4	-	-	34.4±4.3	-	-	128
	2	23.9±7.0	+6.2	0.10	35.6±3.6	+3.5	0.44	131
	4	22.1±6.2	−1.8	0.65	35.0±4.0	+1.7	0.71	122

**∞:** Comparisons are to the same genotype with no drug treatment. p values determined by Student T- test.

# Maximum adult lifespan is the mean lifespan of the 10% of the population that had the longest lifespan.

*: Number of animals observed in three independent trials.

## Discussion

### Chemosensory Neurons Are the Cellular Target of the Lifespan-Extending Drug, Ethosuximide

A variety of drugs have been demonstrated to extend the lifespan of invertebrates, including compounds that are proposed to act as antioxidants [4]–[9], complex chemical mixtures derived from plants [10],[11], resveratrol [12],[13], histone deacetylase inhibitors [63],[64], and compounds that influence the vertebrate nervous system [14]–[17]. In each case, the understanding of how these drugs act to extend lifespan is limited. In particular, the cellular target has not been established for any of these compounds. Advancing this understanding is challenging because of the complexity of the aging phenotype and the fact that many determinants influence lifespan. Characterizing the mechanism of these drugs and defining the cellular target of action is critical for elucidating the endogenous pathways that are affected by the drugs, and ultimately using these drugs in therapeutic applications. Here we demonstrate that chemosensory neurons are the cellular target of ethosuximide in lifespan extension. Two lines of evidence support this conclusion. First, ethosuximide treatment caused the same defects as mutations that result in structural defects in chemosensory neurons, including defective chemotaxis, abnormal dauer arrest, abnormal L1 arrest and extended lifespan. Second, *osm-3* mutants that are defective in a subset of chemosensory neurons were resistant to the lifespan extension caused by ethosuximide treatment and the toxic effects of ethosuximide. Together these results suggest that ethosuximide inhibits chemosensory function to extend *C. elegans* lifespan. This conclusion is important because it defines a specific cellular target for a lifespan extending drug and it demonstrates for the first time that a drug can act on specific cells in the nervous system to extend lifespan.

Our previous studies of the related compound trimethadione demonstrated that *osm-3* mutants are partially resistant to the mean lifespan extension caused by trimethadione and are fully resistant to the maximum lifespan extension cause by trimethadione [14]. These results are consistent with the conclusion that the lifespan extension caused by trimethadione is partly caused by inhibiting the function of chemosensory neurons. The finding that part of the lifespan extending activity of trimethadione was not suppressed by an *osm-3* mutation raises the possibility that trimethadione functions by additional mechanisms to extend lifespan.

We demonstrated that resistance to ethosuximide lethality can be caused by mutations in several different genes that disrupt ciliated neuron structure and function, including *che-3*, *osm-3*, *che-13*, *osm-5* and *daf-10*. Ciliated neurons in the amphid sheath have access to the environment so that they can detect chemicals and function in sensory perception. Because these neurons are exposed to the environment the cell membranes can be stained with lipophilic dyes. These observations raise the possibility that ciliated neurons absorb water-soluble chemicals, like ethosuximide, from the environment and mutations that disrupt cilium structure reduce drug absorption [24]. However, several lines of evidence suggest that mutants with defects in ciliated neurons are normal for drug absorption. First, we demonstrated that *che-3(am162)* mutants were weakly resistant to ethosuximide lethality and strongly defective in DiO staining. By contrast, *che-3(am178)* mutants raised at 20°C were strongly resistant to ethosuximide lethality but displayed relatively normal DiO staining, indicating that the ciliated neurons in these mutants have access to the environment. The lack of a correlation between dye-filling defects and ethosuximide resistance suggests that the ethosuximide resistance of *che-3* mutants is not caused by a defect in the ability of the drug to enter the animal. Second, we demonstrated that *osm-3* mutants are not resistant to drugs in general. Cilium structure mutants are resistant to the effects of the nematicidal drug ivermectin [24]. Therefore, we directly tested the possibility that cilium structure mutants are resistant to drugs in general by analyzing sensitivity to a third compound, serotonin. *osm-3* mutants and wild-type animals displayed similar sensitivity to egg-laying stimulation caused by serotonin, indicating that the mutation does not impair absorption of serotonin. In addition, screens for mutations that cause resistance to fluoxetine [27],[39], levamisole [65], benzimidazole [66], aldicarb [67], nemadipine-A [41], BMS-192364 [40], and α-amanatin [68] have been described. Despite extensive screening and the successful identification of mutants resistant to these compounds, none of these reports describe the isolation of mutations that disrupt the structure of ciliated neurons. While the failure to identify a class of mutations in a genetic screen is not a definitive finding, these results suggest that mutations that disrupt cilium structure do not cause significant resistance to these compounds. Third, we took advantage of our observation that ethosuximide treatment stimulates egg-laying and this effect requires the non-ciliated HSN neurons [14]. *osm-3(p802)* mutants that are highly resistant to the lethality and lifespan extension caused by ethosuximde were nonetheless sensitive to the egg-laying effects of the drug, indicating that these mutants are not defective in ethosuximide absorption. A simple model to explain our findings is that ethosuximide is absorbed by animals independent of the structure of ciliated neurons and the drug influences the activity of multiple neurons throughout the body. The activity of ethosuximide on HSN neurons that mediate egg-laying is intact in *osm-3* mutants whereas the activity on ciliated neurons that mediate toxicity and lifespan extension is altered.

### Chemosensory Neurons Control Lifespan

Chemosensory neurons have been extensively characterized in *C. elegans* to understand behavior, and Kenyon and colleagues demonstrated that chemosensory neurons play an important role in controlling adult lifespan [32],[34]. Mutants that have defects in chemosensory neuron function display an extended lifespan [32], and ablation of specific neurons such as ASI, AWA, and AWC extends the adult lifespan [34]. Here we provide an independent line of evidence that these neurons control lifespan. Our results indicate that ethosuximide inhibits the activity of chemosensory neurons and thereby causes a lifespan extension. These results provide independent support the model that high levels of chemosensory neuronal activity promote a reduced lifespan and inhibiting these neurons causes a lifespan extension.

An important issue that is raised by these observations is how does the activity of chemosensory neurons influence lifespan? One possibility is that the chemosensory neurons regulate the activity of the insulin/IGF-1 signaling pathway. Amphid neurons are the site of expression of many insulin/IGF-1 ligands [69] raising the possibility that these neurons release ligand in response to environmental cues and thereby influence lifespan by an endocrine mechanism. Consistent with this model, ethosuximide enhanced L1 arrest in *daf-2* mutants, similar to *osm-3* mutations [54],[61]. An important test of this model is the dependence of these effects on DAF-16, a transcription factor that mediates effects of insulin/IGF-1 signaling. Ethosuximide enhancement of L1 arrest was dramatically reduced by a *daf-16(lf)* mutation. Furthermore, ablation of ASI amphid neurons extends lifespan in a manner the requires the DAF-16 FOXO transcription factor [34]. These results suggest that amphid neurons are important for insulin/IGF-1 signaling and that ethosuximide may extend lifespan by disrupting this pathway. However, ethosuximide and *osm-3* mutations extend the lifespan of *daf-16* mutants, indicating loss of insulin/IGF-1 signaling cannot fully explain these lifespan extensions [14],[32]. Furthermore, ablation of AWA and AWC amphid neurons extends the lifespan of *daf-16* mutants [34]. Therefore, our data support the model that ethosuximide acts through insulin-dependent and insulin-independent pathways to extend *C. elegans* lifespan.

### Sensory Perception of Food May Accelerate Lifespan, and Inhibition of Sensory Perception May Be Sufficient to Extend Lifespan

Restricting dietary intake can extend lifespan in many animal models [70]–[73], and these observations document the critical role of food availability in controlling lifespan. An important question is how do animals assess food availability to control lifespan? In particular, is food availability determined metabolically, by monitoring nutrients that are ingested, or is food availability determined by neural sensation, by monitoring food derived cues in the environment? Recent studies in *Drosophila* indicate that the effects of dietary restriction are mediated, in part, by the perception of food-derived cues [74]. Exposure of flies to food-derived odors can partially suppress the lifespan extension caused by dietary restriction. Therefore, mutations that extend lifespan by disrupting chemosensation may block perception of food-related chemical cues and thereby activate pathways that respond to dietary restriction.

Here we demonstrate that the anticonvulsant ethosuximide extends *C. elegans* lifespan by inhibiting sensory neurons that are hypothesized to mediate attraction to environmental food sources. Hermaphrodites treated with ethosuximide appear to ingest a normal amount of food since they do not display a diminished body size or progeny production, the characteristics of dietary restricted animals [72]. These results suggest that inhibiting the sensation of food is sufficient to extend lifespan even in the presence of normal food ingestion. These observations raise the exciting possibility that inhibiting the sensation of food may be a conserved mechanism of lifespan extension. In addition to controlling seizures, a common side effect of ethosuximide treatment in humans is loss of taste sensation [75]. Therefore, targeting sensory mediated processes with drugs like ethosuximide may present a means to extend mammalian lifespan.

## Materials and Methods

### General Strains and Methods

*C. elegans* strains were cultured at 20°C on 6 cm Petri dishes containing nematode growth media (NGM) agar and a lawn of *E. coli* strain OP50 unless otherwise noted [76]. Unless otherwise noted, preparation and storage of dishes containing pharmacological compounds was performed as previously described [14].

We used the following *C. elegans* mutations that are described in Riddle *et al.* [23] or in this study: *che-3(e1124)* I, *che-3(am165)* I, *che-3(am162)* I, *che-3(am178)* I, *che-13(e1805)* I, *daf-16(mu86)* I, *unc-11(e47)* I, *dpy-5(e61)* I, *unc-29(e1072)* I, *unc-75(e950)* I, *daf-2(e1370)* III, *daf-2(m41)* III, *osm-3(p802)* IV, *osm-3(am161)* IV, *osm-3(am177)* IV, *osm-3(am172)* IV, *daf-10(e1387)* IV, *osm-5(p813)* X, *lin-15(n765)* X, *cca-1(ad1650)* X, and *cca-1(gk30)* X.

The following well-characterized mutations were used in this study: *che-3(e1124 Q2233stop)* is a probable null mutation in the CHE-3 dynein [42]; *che-13(e1805 Q219stop)* is a loss-of-function mutation that affects the IFT57 protein [77]; *osm-3(p802 Q346stop)* is a strong loss-of-function mutation that disrupts the OSM-3 kinesin [47]; *daf-10(e1387 Q892stop)* is a loss-of-function mutation that disrupts the IFT122 protein [78]; *osm-5(p813 Q473stop)* is a loss-of-function mutation that disrupts the Tg737/Polaris protein [79]; *daf-2(e1370 P1465S)* is a partial loss-of-function mutation that affects the kinase domain of the DAF-2 receptor tyrosine kinase [56]; *daf-2*(*m41* G383E) is a weak loss-of-function mutation that affects the ligand-binding domain of the DAF-2 receptor tyrosine kinase [80]; *daf-16(mu86)* is a probable null mutation that results in deletion of most of the DAF-16 coding sequence including all of the forkhead domain [57]; *cca-1(ad1650)* and *cca-1(gk30)* are strong loss-of-function mutations that result in deletions of different portions of the CCA-1 coding sequence [37].

### Isolation and Genetic Analysis of Ethosuximide­Resistant Mutants

N2 hermaphrodites (P_0_) were mutagenized as described by Brenner [76] with either 50 mM EMS or 0.5 mM ENU. 100,000 F1 hermaphrodites were treated with hypochlorite, and F2 eggs were plated on NGM containing 10–12 mg/ml ethosuximide (Sigma, St. Louis, MO). Since *E. coli* failed to form a thick lawn on dishes containing 12 mg/ml ethosuximide, these dishes were seeded with *E. coli* OP50 that had been concentrated 10-fold. F2 progeny that matured to the L4/adult stage were picked as ethosuximide resistant mutants, and populations derived from these individuals were retested for ethosuximide resistance. 48 independently derived ethosuximide resistant mutants were isolated. These mutant strains were backcrossed at least twice to wild-type (N2) to remove extraneous mutations. Forty-two mutant strains were backcrossed successfully, indicating that ethosuximide resistance was caused by a single mutation.

### Positioning Mutations Relative to SNP Markers and Visible Markers

Single nucleotide polymorphism (SNP) mapping analysis was performed on a subset of resistant mutants by mating ethosuximide resistant mutants (P_0_) to the divergent CB4856 strain (P_0_), selecting F1 outcross progeny, and scoring F2 self-progeny for ethosuximide resistance. F2 animals that displayed resistance were judged to be homozygous for the mutation, and F3 progeny were harvested for DNA. SNPs distributed throughout the *C. elegans* genome [81] were scored using Pyrosequencing (Biotage Foxboro, MA) or direct DNA sequencing. Linkage values were calculated by determining the ratio of N2 DNA to CB4856 DNA at each polymorphism.

The *am178* mutation was tightly linked to a SNP marker at the center of Chromosome I. Three factor mapping experiments with visible markers yielded the following results. From *am178/unc-11 dpy-5* hermaphrodites, 5/6 Unc non Dpy self progeny segregated *am178* and 0/9 Dpy non Unc self progeny segregated *am178*; From *am178/dpy-5 unc-75* hermaphrodites, 3/8 Unc non Dpy self progeny segregated *am178*; From *am178/dpy-5 unc-29* hermaphrodites, 7/8 Dpy non Unc self progeny segregated *am178*. These results position *am178* right of *dpy-5*, left of *unc-75*, and probably left of *unc-29*.

For high resolution SNP mapping of *am178*, we mated *dpy-5(e61) am178* and *am178 unc-75(e950)* homozygotes to CB4856 males, picked F1 outcross progeny, and selected non-Dpy and non-Unc F2 self-progeny resistant to 12 mg/ml ethosuximide. We prepared DNA from strains homozygous for the recombinant chromosome and scored SNP markers.

### Complementation and Transgenic Rescue

Because *che-3* and *osm-3* mutations interfere with male mating, *che-3(e1124)* and *osm-3(p802)* were maintained over *hT2* and *nT1* myo-2::GFP balancer chromosomes, respectively. For complementation analysis with *am178* and *che-3(e1124)*, one *am178* hermaphrodite was mated to five *che-3(e1124)/hT2* males and outcross progeny were scored for ethosuximide resistance. Non-GFP, outcrossed *am178/che-3(e1124)* animals survived 12 mg/ml ethosuximide treatment, indicating that *che-3(e1124)* and *am178* fail to complement for ethosuximide resistance. Neither *che-3(e1124)/+* nor *am178/+* animals survived 12 mg/ml ethosuximide treatment.

For complementation analysis with dye-filling defective alleles, ethosuximide resistant mutants that displayed a Dyf phenotype were mated to either *che-3(e1124)/hT2* or *osm-3(p802)*/nT1 males, and non-GFP outcross progeny were subjected to dye-filling analysis. 9/9 *am172/osm-3(p802)* animals, 15/15 *am177/osm-3(p802)* animals and 20/25 *am161/osm-3(p802)* animals displayed a Dyf phenotype, indicating that these three alleles fail to complement *osm-3(p802)* for dye-filling. 14/15 *am165/ che-3(e1124)* animals and 9/9 *am162/ che-3(e1124)* animals displayed a Dyf phenotype, indicating that these two alleles fail to complement *che-3(e1124)* for dye-filling.

To analyze the ability of genomic DNA to rescue the mutant phenotype of *am178*, we generated transgenic animals containing extrachromosomal arrays using standard procedures [82]. Fosmid clone WRM0637cB0 contains the entire *che-3* coding region and only one other entire gene, F18C12.4. To identify transgenic animals, we co-injected the fosmid clone WRM062bF09 that rescues the *lin-15(n765)* multi-vulval (Muv) phenotype (M. Nonet, personal communication). These two fosmids were co-injected at a concentration of 20 ng/µl each into *am178*; *lin-15(n765ts)* animals to generate the transgenic array *amEx100*. Hermaphrodites containing the *amEx100* array transmitted it to about 73% of self-progeny, since 27% of self-progeny were Muv (n = 100). When self-progeny of *am178; lin-15(n765);amEx100* hermaphrodites were plated on media containing 12 mg/ml ethosuximide, the only animals that survived to adulthood after 5–6 days were Muv (N = 59), indicating that they did not contain *amEx100*. These results indicate that the *amEx100* array rescues the *am178* resistance to ethosuximide phenotype, suggesting that the gene affected by *am178* is contained in fosmid WRM0637cB0.

### DNA Sequence Analysis and RNA Analysis

DNA sequencing was performed using standard procedures. We PCR amplified predicted exons and splice junctions (www.wormbase.org) from the *che-3* and *osm-3* genes using DNA from *am178* and *am177* mutant animals, respectively. The *am178* strain contained a G to A change at the fifth base of intron 9. To analyze the *che-3* messenger RNA, we prepared RNA from a population of *am178* mutants (TRizol, Invitrogen), DNase treated the RNA (DNA-free, Ambion), and generated cDNA by performing reverse transcription (Retroscript, Ambion). We PCR amplified the junctions between exons 9 and 10 and determined the DNA sequence of the PCR product to infer the splicing pattern of the mRNA. The *che-3* mRNA derived from *am178* mutants displayed a deletion of 17 nucleotides (GTCAGCTTGGTTTTTGC) in exon 9.

### Dye-Filling Analysis

Staining with DiO was performed using previously described methods [31],[83]. L4 hermaphrodites were placed in 100 µL of M9 buffer containing 20 µg/ml DiO (Molecular Probes) in a microtiter dish and incubated for ∼2 hrs at room temperature. To remove non-specifically bound DiO, we transferred the animals to a NGM dish seeded with *E. coli* OP50 and cultured for 20–30 minutes at room temperature. For analysis of strains in Table 1, five to fifteen animals were observed using a Zeiss Axioplan 2 microscope equipped for fluorescence microscopy at 400× magnification. Animals were categorized in the following classes: (I) No amphid neurons stained; (II) 1–2 amphid neurons stained; or (III) more than 2 amphid neurons stained. All the strains described in Table 1 displayed only the class I or only the class III pattern of staining. For analysis of *che-3(am178)* mutants in Table 2, DiO staining was observed using an Olympus SZX12 dissecting microscope equipped for fluorescence microscopy at 144× magnification. Animals were scored as Dyf if no amphid neurons stained robustly with DiO.

### Quantification of Ethosuximide Resistance

To quantify the penetrance of the ethosuximide resistance phenotype, we adapted an assay method described by Rand and Johnson [84]. Eggs were picked to a Petri dish with agar containing 12 mg/ml ethosuximide and counted. Five to six days later we counted the number of animals that matured past the L1 stage. For resistant strains most animals were L4 larvae or adults. The remaining eggs produced animals that did not mature past the L1 stage or left the agar surface. The number of animals that matured past the L1 stage was divided by the total number of eggs to determine the percent resistant to ethosuximide.

### Chemotaxis to Volatile Compounds

For pharmacological analysis of chemotaxis, dishes were prepared by adding powdered ethosuximide directly to 60°C chemotaxis agar, agitating to dissolve the compound and dispensing to 10 cM Petri dishes. Chemotaxis assays were performed as described previously [85] with two minor modifications. First, to obtain a large number of well-fed animals for the assay, we cultured animals from conception to adulthood in the presence of ethosuximide or no drug on 6 cM Petri dishes containing OP50 that was concentrated 10-fold. Second, we added ethosuximide to the molten chemotaxis agar before dispensing into 10 cM Petri dishes and used these dishes after 3–8 hours. To conduct chemotaxis assays, we pipeted animals in a small volume of liquid onto the center of a 10 cM Petri dish that had been pre-treated with 1 µL of 1M Na azide, a paralyzing agent, at two diametrically opposed spots on the plate. At this time, we added 1 µL of volatile odorant to one azide treated spot and 1 µL of ethanol as a control to the other azide treated spot. Isoamyl alcohol and diacetyl were diluted in ethanol at concentrations of 1∶10 and 1∶1000, respectively. After 60 minutes we scored the number of paralyzed animals at each azide spot and the number of moving animals on the plate. The sodium azide spots were positioned at the edge of the dish, and animals that desiccated on the side of the dish at the positions of the sodium azide spots were attributed to those categories; otherwise such animals were attributed to the moving animal category. Chemotaxis index was determined using the following formula:
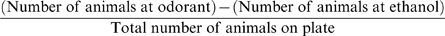



### Analysis of Egg-Laying

The analysis of egg-laying was performed as described previously [85]. To determine the effect of ethosuximide on egg-laying, we picked L4 hermaphrodites, incubated them at 20°C for approximately 20 hours on NGM plates with abundant food, individually placed them in 100 µL of M9 buffer in a microtiter dish plus or minus ethosuximide, and counted the number of eggs laid in 90 minutes. We used a similar method to analyze serotonin, except animals were placed in 50 µL buffer.

### Analysis of Dauer Larvae and Larval Arrest Phenotypes

To analyze dauer formation at 27°C, we allowed 8–10 gravid hermaphrodites to lay eggs on one Petri dish for 4–8 hours at room temperature and then incubated the dish at 27°C for precisely 44 hours [52]. We counted the total number of animals on the dish, flooded the dish with 1% SDS to kill all animals except for dauer larvae, and counted the number of live dauer larvae after 15 minutes. To analyze dauer formation at 25°C, we prepared NGM media without peptone, the main carbon source for the *E. coli* OP50. The omission of peptone from the media allowed us to provide a consistent amount of *E. coli* OP50 food, and made the experiment independent of the effects of ethosuximide on bacterial proliferation. We aliquoted 200 µl of an overnight *E. coli* OP50 culture that had been concentrated 10-fold on each dish. We placed two L4 hermaphrodites on each dish, incubated the animals at 25°C, and monitored the dishes daily for starvation [86]. Five days post-starvation we scored the number of dauer larvae by flooding the dishes with 1% SDS and counting the number of live animals after 15 minutes. Dauer formation of *daf-2(m41)* mutants was analyzed as described previously [16].

To analyze the L1 arrest phenotype, we cultured L4 hermaphrodites 1–2 days on NGM dishes with 4 mg/ml ethosuximide or no drug, transferred these adults to fresh dishes with 4 mg/ml ethosuximide or no drug for 4–8 hours at room temperature, and removed the adults. These dishes containing freshly deposited eggs were cultured at 25.5°C for 48 hours. We counted the number of eggs, L1 larvae, and older larval stages.

### Lifespan Analysis

For a typical lifespan experiment, parental worms were cultured in the presence of the drug, and progeny were selected at the L4 stage for lifespan analysis. Thus, these progeny were exposed to drug from the time of conception until death. For measurements of lifespan, hermaphrodites were chosen for analysis at the L4 stage (defined as day 0) and analyzed every 1–2 days from day 3 until death. Approximately 15 hermaphrodites were cultured on each Petri dish. Hermaphrodites were transferred to fresh Petri dishes about every two days until the cessation of progeny production and about every week thereafter. Animals were scored as dead if they displayed no spontaneous movement or response when prodded. Dead worms that displayed internally hatched progeny, an extruded gonad or desiccation due to crawling off the agar were excluded from the data. Lifespan is the number of days from the L4 stage to the average of the last day a worm was observed to be alive and the first day a worm was observed to be dead. Lifespan experiments involving pharmacological compounds were always done in parallel with a control group.

### Statistical Methods

For each experimental group, comparisons were made to a control group maintained in the same incubator and analyzed at the same time points. Mean, standard error, P values, and other statistical parameters were calculated using InStat 2.03 software (Graphpad Software) or Microsoft Excel.
